# Prevalence of rotator cuff tears: a systematic review and meta-analysis of observational studies

**DOI:** 10.3389/fphys.2026.1876217

**Published:** 2026-09-03

**Authors:** Yaocheng Yang, Xinlei Fu, Shilong Meng, Musen Zhong

**Affiliations:** 1 Xiamen Hospital of Traditional Chinese Medicine, Xiamen, Fujian, China; 2 The First Affiliated Hospital of Zhejiang Chinese Medical University, Hangzhou, Zhejiang, China; 3 The Second Clinical Medical College of Zhejiang Chinese Medical University, Hangzhou, Zhejiang, China; 4 Chunan County Hospital of Traditional Chinese Medicine, Hangzhou, Zhejiang, China

**Keywords:** epidemiology, meta-analysis, prevalence, rotator cuff tear, systematic review

## Abstract

**Background:**

Rotator cuff tears are a major cause of shoulder pain, functional impairment, and reduced quality of life. However, reported prevalence estimates vary substantially across population settings, tear definitions, and diagnostic methods. This study aimed to quantify the prevalence of rotator cuff tears and identify factors contributing to variation across studies.

**Methods:**

We conducted a systematic review and meta-analysis of observational studies reporting the prevalence of rotator cuff tears. CNKI, Wanfang, VIP, PubMed, Web of Science, Embase, and the Cochrane Library were searched from database inception to December 1, 2025. Studies based on ultrasound or magnetic resonance imaging (MRI) and reporting prevalence on a per-person basis were included. Two reviewers independently performed study screening, data extraction, and quality assessment using the Agency for Healthcare Research and Quality (AHRQ) cross-sectional study appraisal tool. Statistical analyses were performed using Stata 18.0 and Python 3.13.

**Results:**

Seventeen studies representing 19 independent datasets and 5,988 participants from eight countries were included. The pooled prevalence was 38.6% *(95% CI*, 29.2%–48.0%; *I²* = 98.5%). Prevalence varied by population source, from 23.6% in asymptomatic or never-presented populations to 55.5% in symptomatic, trauma, or outpatient populations and 57.3% in specific high-exposure groups. The pooled prevalence was lower for full-thickness tears than under an any-tear definition (21.0% vs 43.8%), whereas ultrasound- and MRI-based estimates were similar (37.5% vs 40.5%). *Post hoc* exploratory meta-regression identified population source as the only statistically significant moderator, explaining approximately 62.1% of between-study heterogeneity. Alternative transformations yielded similar pooled estimates, while restricted analyses yielded prevalence estimates of approximately 64% in shoulder-related clinical populations.

**Conclusion:**

Rotator cuff tears are common, particularly among symptomatic and high-exposure populations, underscoring the need for targeted prevention, risk assessment, and early detection. The marked variation across population settings further highlights the importance of population-specific prevalence estimates for clinical and epidemiological planning.**Systematic Review Registration:** https://www.crd.york.ac.uk/PROSPERO/, identifier CRD420251251902.

## 1 Introduction

Rotator cuff tears (RCTs) are common shoulder disorders characterized by partial- or full-thickness disruption of the rotator cuff tendons. They are a major cause of shoulder pain and functional impairment (Bedi et al., 2024). Their etiology is multifactorial and may involve acute trauma, chronic impingement, tendon degeneration, and age-related degenerative changes (Zhao et al., 2023; Liu et al., 2024; Albishi et al., 2025). Patients commonly present with restricted shoulder motion, muscle weakness, and nocturnal pain, and in severe cases may develop muscle atrophy and joint stiffness, substantially impairing quality of life and daily functioning (Altamimi et al., 2024).

The diagnosis of rotator cuff tears is generally based on clinical history, physical examination, and imaging studies, with ultrasound and magnetic resonance imaging (MRI) being the most commonly used modalities (Zoga et al., 2021; Ye et al., 2026). Previous studies suggest that the prevalence of rotator cuff tears increases with age and is particularly high among middle-aged and older adults (Tashjian, 2012; Jain and Khazzam, 2024). Moreover, imaging studies have shown that rotator cuff tears can also be detected in asymptomatic individuals (Sanders et al., 2025). These findings indicate that rotator cuff tears are not confined to clinically symptomatic populations and that epidemiological estimates may be influenced by multiple factors, including age, population source, and study setting.

However, published estimates of rotator cuff tear prevalence vary considerably across studies (Teunis et al., 2014; Bigna, 2026). Differences in population source, age distribution, imaging modality, and tear definition make direct comparisons difficult and limit a clear understanding of the epidemiological profile of rotator cuff tears. In addition, existing evidence syntheses have mainly focused on rotator cuff disease or imaging abnormalities rather than on the prevalence of rotator cuff tears themselves across different research settings. Systematic comparisons across population sources, tear definitions, and diagnostic modalities remain limited.

In this study, we conducted a systematic review and meta-analysis of observational studies to estimate the prevalence of RCTs and examine variation across subgroups defined by population source, age, sex, geographic region, tear definition, and imaging modality. We further aimed to characterize the epidemiological distribution of RCTs and provide evidence to inform population-specific risk assessment, early identification, and healthcare planning.

## 2 Methods

This systematic review and meta-analysis was conducted in accordance with the Preferred Reporting Items for Systematic Reviews and Meta-Analyses (PRISMA) 2020 statement (Page et al., 2021). The review was registered in PROSPERO on December 11, 2025 (CRD420251251902).

### 2.1 Search strategy

We systematically searched CNKI, Wanfang, VIP, PubMed, Web of Science, Embase, and the Cochrane Library from database inception to December 1, 2025. No language restrictions were applied. The search strategy combined controlled vocabulary terms and free-text terms, and was adapted as appropriate for each database. The search concepts included rotator cuff tear, rotator cuff injury, rotator cuff pathology, rotator cuff disease, prevalence, and epidemiology. For the Chinese databases, the corresponding Chinese search terms were used.

To minimize the risk of missing eligible studies, we also manually screened the reference lists of the included studies and relevant reviews. The complete PubMed search strategy was as follows: (“Rotator Cuff”[Mesh] OR “Rotator Cuff Injuries”[Mesh] OR “rotator cuff”[tiab] OR “rotator cuff tear”[tiab] OR “rotator cuff tears”[tiab] OR “rotator cuff injury”[tiab] OR “rotator cuff injuries”[tiab] OR “rotator cuff disease”[tiab] OR “rotator cuff diseases”[tiab] OR “rotator cuff pathology”[tiab] OR “rotator cuff pathologies”[tiab]) AND (“Prevalence”[Mesh] OR “Epidemiology”[Subheading] OR prevalen*[tiab] OR epidemiolog*[tiab]). The full search strategies for all databases are provided in **Supplementary File 1**.

### 2.2 Inclusion and exclusion criteria

The eligibility criteria were developed according to the CoCoPop framework, which comprises the Condition, Context, and Population of interest (Munn et al., 2015; Munn et al., 2018).

Population: Eligible participants were individuals who underwent shoulder imaging with ultrasound or magnetic resonance imaging (MRI). No restrictions were placed on population source; general-population or community-based samples, clinical populations, and specific occupational or high-exposure groups were all eligible.

Context: Studies conducted in any country, healthcare setting, or research setting were eligible. Population source and study setting were subsequently considered in subgroup analyses.

Condition: The condition of interest was a rotator cuff tear diagnosed using clearly defined ultrasound or MRI findings (Zoga et al., 2021; Toh, 2024). No restrictions were placed on tear thickness or the specific rotator cuff tendon involved.

Inclusion criteria:

(1) the study population underwent shoulder ultrasound or MRI; (2) the study reported the prevalence of rotator cuff tears, or the prevalence could be calculated from the original data; (3) the diagnosis of rotator cuff tears was clearly defined and based on ultrasound or MRI findings; (4) the study was observational in design, including cross-sectional studies, retrospective studies, or prospective cohort studies from which baseline prevalence data could be extracted; (5) prevalence data were reported on a per-person basis or could be converted to a per-person prevalence from the original data.

Exclusion criteria:

(1) duplicate publications; (2) case reports, case series, conference abstracts, reviews, commentaries, or other non-original studies; (3) the prevalence of rotator cuff tears could not be extracted or calculated; (4) the shoulder was used as the statistical unit and the data could not be converted to a per-person basis; (5) ultrasound or MRI was not used as the diagnostic basis; (6) diagnostic criteria were unclear, or key data were seriously incomplete and could not be obtained from the available report.

### 2.3 Literature screening and data extraction

The retrieved records were imported into EndNote X9.1 for reference management and duplicate removal. Two reviewers independently screened the titles and abstracts for initial eligibility. Full-text articles considered potentially relevant were then retrieved and assessed against the predefined inclusion and exclusion criteria. Any disagreements were resolved through discussion or, when necessary, consultation with a third reviewer.

Two reviewers independently extracted data using a standardized data extraction form. Extracted variables included first author, year of publication, study region, study design, population source, sample size, number of participants with rotator cuff tears, age, sex, diagnostic modality, tear definition, study period, and information required for quality assessment.

All analyses were conducted on a per-person basis. For studies reporting both the number of shoulders and the number of participants, participant-level prevalence data were extracted. For studies assessing unilateral, bilateral, or dominant-side shoulders, data were included in the quantitative synthesis only when prevalence could be determined on a per-person basis.

If a single study reported two or more independent, non-overlapping population samples, each sample was treated as a separate dataset in the meta-analysis. In contrast, when a study reported multiple mutually exclusive subgroup estimates, such as age- or sex-specific strata, these data were used only in the corresponding subgroup analyses and were not double-counted in the overall analysis.

Studies were included in each quantitative analysis only when the required data were available, and missing subgroup-level variables were not imputed. After data extraction, the two reviewers cross-checked all extracted items, and any discrepancies were resolved through discussion or consultation with a third reviewer.

### 2.4 Assessment of study quality

Methodological quality was assessed using the Agency for Healthcare Research and Quality (AHRQ) checklist for cross-sectional studies (Ma et al., 2020; Mamikutty et al., 2021). This instrument contains 11 items covering data sources, participant selection, inclusion and exclusion criteria, control of confounding, handling of missing data, response rates, and follow-up. Each item was scored as 1 for “Yes” and 0 for “No” or “Unclear,” yielding a total score ranging from 0 to 11. Studies scoring 8–11 were considered high quality, 4–7 moderate quality, and 0–3 low quality.

Because most included studies were cross-sectional or provided prevalence data that could be evaluated in a cross-sectional manner, the AHRQ checklist was considered appropriate for this review. Quality assessment was performed independently by two reviewers. Any disagreements were resolved through discussion, and if necessary, adjudicated by a third reviewer. Quality assessment results were not used as the sole basis for study inclusion, but were considered in the interpretation of findings and the overall assessment of evidence reliability. The detailed checklist items are shown in **Table 1**.

**Table 1:** AHRQ checklist for cross-sectional studies.

No.	Item content	Yes	No/Unclear	
1	Define the source of information (survey or record review)?			
2	List inclusion and exclusion criteria for exposed and unexposed subjects (cases and controls) or refer to previous publications			
3	Indicate the period used for identifying patients			
4	Indicate whether or not subjects were consecutive if not population-based			
5	Indicate if evaluators of subjective components of the study were masked to other aspects of the status of the participants			
6	Describe any assessments undertaken for quality assurance purposes (e.g., test/retest of primary outcome measurements)			
7	Explain any patient exclusions from the analysis			
8	Describe how confounding was assessed and/or controlled			
9	If applicable, explain how missing data were handled in the analysis			
10	Summarize patient response rates and completeness of data collection			
11	Clarify what follow-up, if any, was expected and the percentage of patients for which incomplete data or follow-up was obtained		

### 2.5 Statistical methods

Statistical analyses were performed using Stata 18.0 and Python 3.13. In the primary analysis, raw prevalence estimates were pooled without variance-stabilizing transformation. Sampling variances were calculated using the binomial distribution, and pooled estimates with 95% confidence intervals were obtained by inverse-variance weighting. Random-effects models used the DerSimonian–Laird estimator. Heterogeneity was assessed using Cochran’s *Q* test and the *I²* statistic, with *P* < 0.10 indicating statistically significant heterogeneity (Maitra, 2025). A random-effects model was used for the overall analysis. For subgroup analyses, fixed-effect models were applied when within-subgroup heterogeneity was not statistically significant; otherwise, random-effects models were used. Subgroup analyses were conducted according to sex, age, geographic region, study period, population source, tear definition, and imaging modality. When the participant recruitment or data-collection period was unavailable, publication year was used to classify study period.

Robustness was assessed through leave-one-dataset-out sensitivity analysis. *Post hoc* analyses included univariable meta-regression, restricted analyses of clinically defined populations, alternative effect-size transformations, re-estimation of random-effects models using restricted maximum likelihood, and assessment of small-study effects. Meta-regression examined publication year, population source, imaging modality, geographic region, and AHRQ quality score. To reduce the risk of overfitting, only univariable models were fitted. These models used restricted maximum likelihood estimation with Knapp–Hartung adjustment, and pseudo-R² was used to quantify the proportion of between-study heterogeneity explained. Restricted analyses included patients presenting with shoulder pain or acute shoulder trauma and studies explicitly enrolling patients because of shoulder pain.

Sensitivity analyses using logit and Freeman–Tukey double-arcsine transformations were back-transformed to the prevalence scale, with the harmonic mean of study sample sizes used for Freeman–Tukey estimates. Small-study effects were evaluated using a random-effects Egger-type meta-regression on the logit-transformed prevalence scale with restricted maximum likelihood and Knapp–Hartung adjustment, supplemented by traditional Egger regression and the Begg–Mazumdar rank correlation test. A two-sided *P* value < 0.05 was considered statistically significant, except for heterogeneity testing.

## 3 Results

### 3.1 Literature screening process and results

**Figure 1:**
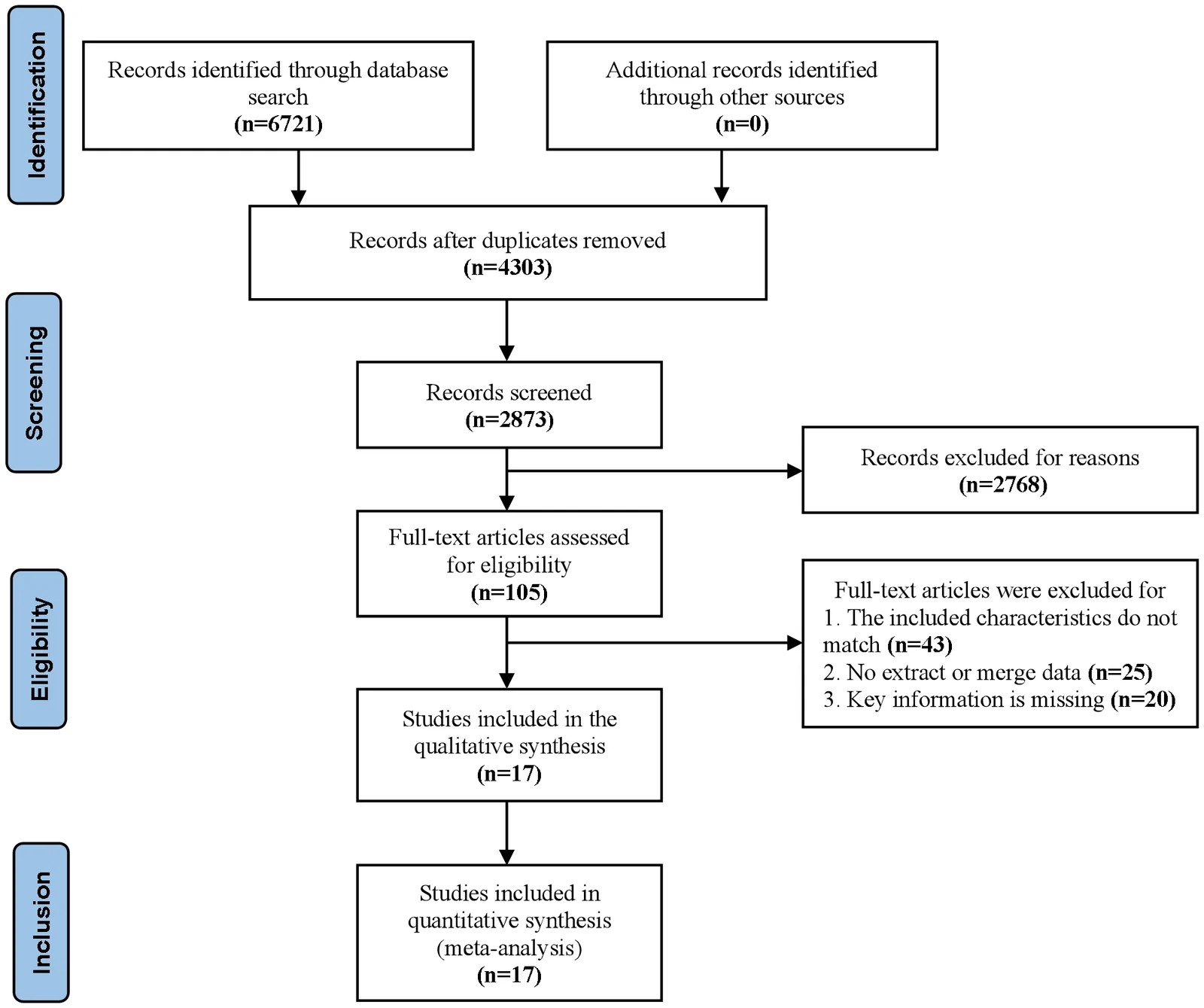
Flow diagram of study identification and selection.

A total of 6,721 records were identified through database searching. After removal of duplicates, the remaining records were screened by title and abstract, and potentially eligible articles were retrieved for full-text assessment. Following full-text review, 17 studies (Tempelhof et al., 1999; Yamaguchi et al., 2006; Sørensen et al., 2007; Fehringer et al., 2008; Kim et al., 2009; Akbar et al., 2010; Baumgarten et al., 2010; Abate et al., 2014; Mcmahon et al., 2014; Jeong et al., 2017; Moon et al., 2018; Park et al., 2018; Kim et al., 2019; Loew et al., 2019; Khoschnau et al., 2020; Harada et al., 2022; Hinsley et al., 2022) were included in the final analysis. The study selection process and reasons for exclusion are presented in **Figure 1**.

### 3.2 Basic characteristics of the included studies and results of the quality assessment

The 17 included studies (Tempelhof et al., 1999; Yamaguchi et al., 2006; Sørensen et al., 2007; Fehringer et al., 2008; Kim et al., 2009; Akbar et al., 2010; Baumgarten et al., 2010; Abate et al., 2014; Mcmahon et al., 2014; Jeong et al., 2017; Moon et al., 2018; Park et al., 2018; Kim et al., 2019; Loew et al., 2019; Khoschnau et al., 2020; Harada et al., 2022; Hinsley et al., 2022), published between 1999 and 2022, represented 19 independent datasets involving 5,988 participants from 8 countries. Study populations included asymptomatic or community-screening participants, symptomatic/trauma/outpatient populations, and specific high-exposure groups. Quality assessment showed that 7 studies (Tempelhof et al., 1999; Yamaguchi et al., 2006; Sørensen et al., 2007; Akbar et al., 2010; Baumgarten et al., 2010; Park et al., 2018; Hinsley et al., 2022) were rated as high quality and 10 (Fehringer et al., 2008; Kim et al., 2009; Abate et al., 2014; Mcmahon et al., 2014; Jeong et al., 2017; Moon et al., 2018; Kim et al., 2019; Loew et al., 2019; Khoschnau et al., 2020; Harada et al., 2022) as moderate quality, with no studies rated as low quality. The main methodological limitations were incomplete reporting of the study period, assessor blinding, missing-data handling, and follow-up information, where applicable. The characteristics of the included studies and quality assessment results are summarized in **Table 2**, and detailed study-level scoring results are provided in **Supplementary File 2**.

**Table 2:** Characteristics of included studies and quality assessment results.

No.	Study	Country	Population	Imaging modality	Cases, n	Participants, n	AHRQ score
1	Tempelhof et al. (1999)	Germany	Asymptomatic volunteers	Ultrasound	96	411	9
2	Yamaguchi et al. (2006)	United States	Patients with unilateral shoulder pain	Ultrasound	376	588	8
3	Sørensen et al. (2007)	Denmark	Patients with acute shoulder injuries	Ultrasound	60	104	10
4	Fehringer et al. (2008)	United States	Outpatients aged ≥65 years with lower-limb orthopedic conditions and no prior shoulder surgery	Ultrasound	35	104	5
5	Kim et al. (2009)	United States	Asymptomatic volunteers	Ultrasound	41	237	5
6a	Akbar et al. (2010)①	Germany	Long-term wheelchair-dependent paraplegic patients	MRI	63	100	8
6b	Akbar et al. (2010)②	Germany	General population controls	MRI	15	100	8
7	Baumgarten et al. (2010)	United States	Adults aged ≥18 years with unilateral, non-traumatic shoulder pain and no prior shoulder surgery	Ultrasound	375	586	9
8	Abate et al. (2014)	Italy	Community-dwelling postmenopausal women without shoulder pain or functional impairment	Ultrasound	27	232	4
9	Mcmahon et al. (2014)	United States	Senior athletes and volunteers aged ≥60 years	Ultrasound	98	141	6
10	Jeong et al. (2017)	South Korea	Volunteers without shoulder symptoms	Ultrasound	105	486	7
11	Moon et al. (2018)	South Korea	Rural community residents aged ≥40 years	MRI	527	987	6
12	Park et al. (2018)	South Korea	Rural community volunteers	MRI	199	634	8
13	Kim et al. (2019)	South Korea	Full-time fruit farmers	MRI	275	455	7
14a	Loew et al. (2019)①	Germany	Full-time painters (male; ≥10 years of continuous employment)	MRI	52	100	7
14b	Loew et al. (2019)②	Germany	General population controls	MRI	9	100	7
15	Khoschnau et al. (2020)	Sweden	Volunteers who had never sought medical care for shoulder problems	Ultrasound	32	106	5
16	Harada et al. (2022)	Japan	Recreational senior tennis players aged ≥60 years	Ultrasound	19	53	6
17	Hinsley et al. (2022)	United Kingdom	Middle-aged and older women	Ultrasound	103	464	10

The 17 included studies contributed 19 independent datasets. Akbar et al. and Loew et al. each reported 2 independent samples and are therefore presented separately in this table. Cases, n, refers to the number of participants with rotator cuff tears. AHRQ scores were assigned at the study level; for studies contributing more than 1 dataset, the same study-level score is shown for each dataset. MRI, magnetic resonance imaging; AHRQ, Agency for Healthcare Research and Quality.

### 3.3 Results of the meta-analysis

#### 3.3.1 Meta-analysis of the prevalence of rotator cuff tears

**Figure 2:**
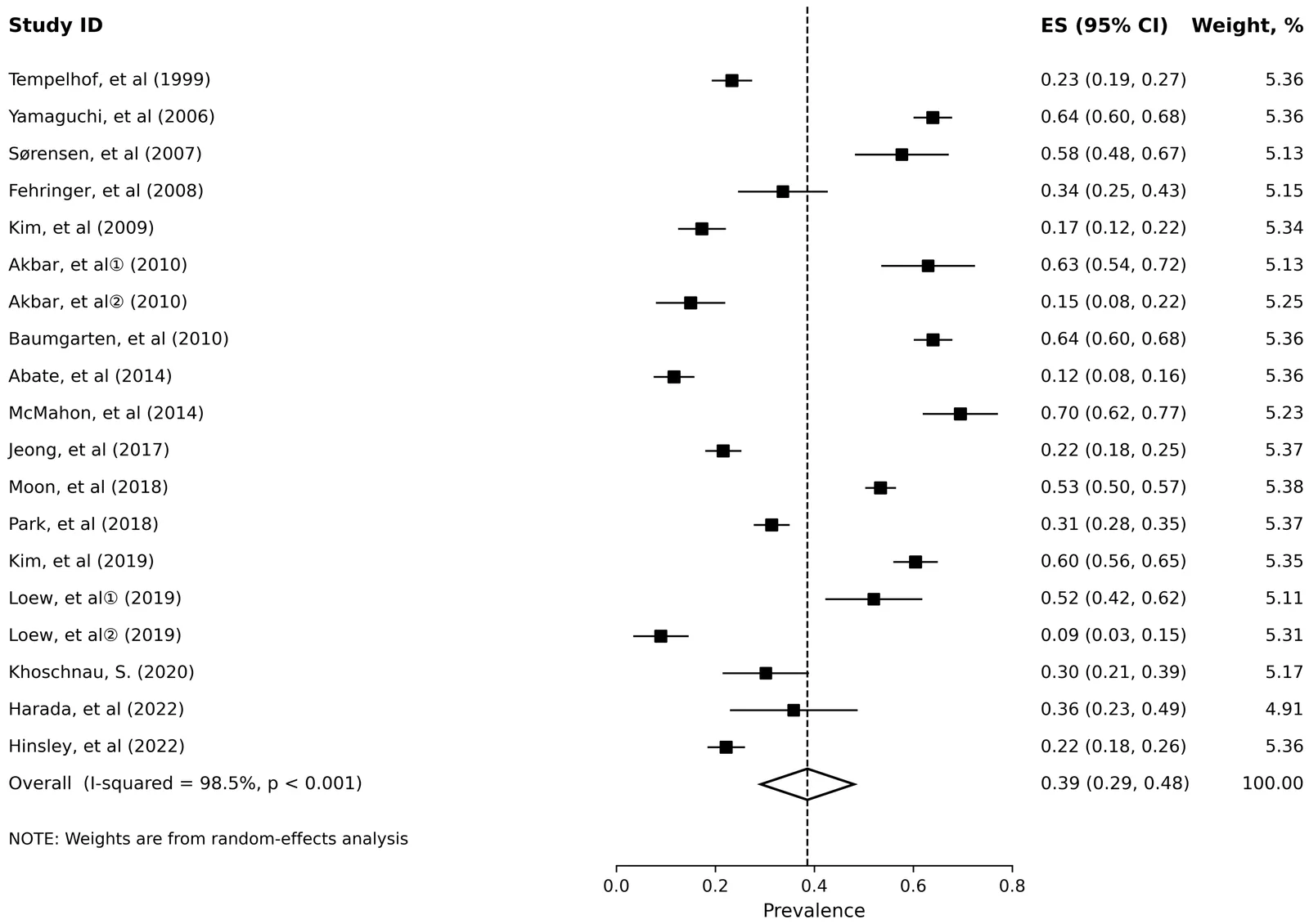
Forest plot of the pooled prevalence of rotator cuff tears.

Seventeen studies (Tempelhof et al., 1999; Yamaguchi et al., 2006; Sørensen et al., 2007; Fehringer et al., 2008; Kim et al., 2009; Akbar et al., 2010; Baumgarten et al., 2010; Abate et al., 2014; Mcmahon et al., 2014; Jeong et al., 2017; Moon et al., 2018; Park et al., 2018; Kim et al., 2019; Loew et al., 2019; Khoschnau et al., 2020; Harada et al., 2022; Hinsley et al., 2022) were included, and 2 (Akbar et al., 2010; Loew et al., 2019) of them each contributed 2 independent samples, resulting in 19 datasets for meta-analysis. Substantial between-study heterogeneity was observed (*I²* = 98.5%, *P* < 0.001); therefore, a random-effects model was applied. The pooled prevalence of rotator cuff tears was 38.6% (*95% CI*, 29.2%–48.0%). The forest plot is shown in **Figure 2**, and the corresponding summary results are presented in **Table 3**.

**Table 3:** Subgroup meta-analysis of rotator cuff tear prevalence.

Subgroup	No. of contributing studies	Heterogeneity		Effect model	Pooled prevalence, % *(95% CI)*
		*I* ^2^	*P*		
Overall prevalence (Tempelhof et al., 1999; Yamaguchi et al., 2006; Sørensen et al., 2007; Fehringer et al., 2008; Kim et al., 2009; Akbar et al., 2010; Baumgarten et al., 2010; Abate et al., 2014; Mcmahon et al., 2014; Jeong et al., 2017; Moon et al., 2018; Park et al., 2018; Kim et al., 2019; Loew et al., 2019; Khoschnau et al., 2020; Harada et al., 2022; Hinsley et al., 2022)	17 (19 datasets)	98.5%	< 0.001	Random-effects model	38.6 (29.2, 48.0)
Sex					
Male (Tempelhof et al., 1999; Kim et al., 2009; Jeong et al., 2017; Moon et al., 2018; Park et al., 2018; Loew et al., 2019; Harada et al., 2022)	7	96.9%	< 0.001	Random-effects model	32.4 (19.5, 45.4)
Female (Tempelhof et al., 1999; Kim et al., 2009; Abate et al., 2014; Jeong et al., 2017; Moon et al., 2018; Park et al., 2018; Harada et al., 2022; Hinsley et al., 2022)	8	96.4%	< 0.001	Random-effects model	24.2 (14.7, 33.7)
Age group, years					
40–49 (Sørensen et al., 2007; Kim et al., 2009; Park et al., 2018)	3	16.6%	0.274	Fixed-effect model	18.8 (10.6, 26.9)
50–59 (Tempelhof et al., 1999; Sørensen et al., 2007; Kim et al., 2009; Jeong et al., 2017; Moon et al., 2018; Park et al., 2018)	6	98.0%	< 0.001	Random-effects model	23.7 (8.7, 38.8)
60–69 (Tempelhof et al., 1999; Sørensen et al., 2007; Kim et al., 2009; Jeong et al., 2017; Moon et al., 2018; Park et al., 2018; Harada et al., 2022; Hinsley et al., 2022)	8	96.6%	< 0.001	Random-effects model	29.7 (15.1, 44.2)
70–79 (Tempelhof et al., 1999; Harada et al., 2022; Hinsley et al., 2022)	3	42.2%	0.177	Fixed-effect model	28.3 (23.5, 33.1)
80–89 (Harada et al., 2022; Hinsley et al., 2022)	2	0.0%	0.412	Fixed-effect model	29.9 (19.5, 40.4)
Region					
North America (Yamaguchi et al., 2006; Fehringer et al., 2008; Kim et al., 2009; Baumgarten et al., 2010; Mcmahon et al., 2014)	5	98.7%	< 0.001	Random-effects model	49.7 (29.5, 70.0)
Europe (Tempelhof et al., 1999; Sørensen et al., 2007; Akbar et al., 2010; Abate et al., 2014; Loew et al., 2019; Khoschnau et al., 2020; Hinsley et al., 2022)	7	96.3%	< 0.001	Random-effects model	31.0 (20.9, 41.1)
East Asia (Jeong et al., 2017; Moon et al., 2018; Park et al., 2018; Kim et al., 2019; Harada et al., 2022)	5	98.5%	< 0.001	Random-effects model	40.6 (25.2, 56.0)
Study period					
Before 2010 (Tempelhof et al., 1999; Yamaguchi et al., 2006; Sørensen et al., 2007; Fehringer et al., 2008; Kim et al., 2009; Akbar et al., 2010; Baumgarten et al., 2010; Mcmahon et al., 2014; Khoschnau et al., 2020; Hinsley et al., 2022)	10	98.5%	< 0.001	Random-effects model	41.7 (28.2, 55.3)
2010 and later (Abate et al., 2014; Jeong et al., 2017; Moon et al., 2018; Park et al., 2018; Kim et al., 2019; Loew et al., 2019; Harada et al., 2022)	7	98.7%	< 0.001	Random-effects model	34.3 (20.3, 48.4)
Population source					
Asymptomatic or never-presented populations (Tempelhof et al., 1999; Kim et al., 2009; Akbar et al., 2010; Abate et al., 2014; Jeong et al., 2017; Moon et al., 2018; Park et al., 2018; Loew et al., 2019; Khoschnau et al., 2020; Hinsley et al., 2022)	10	97.9%	< 0.001	Random-effects model	23.6 (14.2, 32.9)
Symptomatic/trauma/outpatient populations (Yamaguchi et al., 2006; Sørensen et al., 2007; Fehringer et al., 2008; Baumgarten et al., 2010)	4	92.5%	< 0.001	Random-effects model	55.5 (45.2, 65.8)
Specific high-exposure groups (Akbar et al., 2010; Mcmahon et al., 2014; Kim et al., 2019; Loew et al., 2019; Harada et al., 2022)	5	82.2%	< 0.001	Random-effects model	57.3 (48.6, 65.9)
Tear definition					
Full-thickness tear (Tempelhof et al., 1999; Yamaguchi et al., 2006; Sørensen et al., 2007; Fehringer et al., 2008; Kim et al., 2009; Akbar et al., 2010; Baumgarten et al., 2010; Abate et al., 2014; Mcmahon et al., 2014; Jeong et al., 2017; Moon et al., 2018; Park et al., 2018; Loew et al., 2019; Khoschnau et al., 2020; Hinsley et al., 2022)	15	97.5%	< 0.001	Random-effects model	21.0 (15.2, 26.8)
Any tear (Yamaguchi et al., 2006; Sørensen et al., 2007; Kim et al., 2009; Akbar et al., 2010; Baumgarten et al., 2010; Mcmahon et al., 2014; Jeong et al., 2017; Moon et al., 2018; Park et al., 2018; Kim et al., 2019; Loew et al., 2019; Harada et al., 2022)	12	98.5%	< 0.001	Random-effects model	43.8 (32.8, 54.9)
Imaging modality					
Ultrasound (Tempelhof et al., 1999; Yamaguchi et al., 2006; Sørensen et al., 2007; Fehringer et al., 2008; Kim et al., 2009; Baumgarten et al., 2010; Abate et al., 2014; Mcmahon et al., 2014; Jeong et al., 2017; Khoschnau et al., 2020; Harada et al., 2022; Hinsley et al., 2022)	12	98.7%	< 0.001	Random-effects model	37.5 (24.9, 50.1)
MRI (Akbar et al., 2010; Moon et al., 2018; Park et al., 2018; Kim et al., 2019; Loew et al., 2019)	5	98.3%	< 0.001	Random-effects model	40.5 (25.6, 55.4)

No. of contributing studies indicates the number of studies contributing data to each subgroup. The overall pooled estimate was based on 19 independent datasets contributed by 17 studies; two studies each provided 2 independent samples. Specific high-exposure groups included farmers, painters, paraplegic patients, and athletes. CI, confidence interval; MRI, magnetic resonance imaging.

#### 3.3.2 Subgroup analysis of the prevalence of rotator cuff tears

Given the substantial heterogeneity observed in the overall meta-analysis (*I²* = 98.5%, *P* < 0.001), subgroup analyses were conducted according to sex, age group, geographic region, study period, population source, tear definition, and imaging modality to explore potential sources of heterogeneity. The pooled prevalence estimates differed across subgroups, particularly by population source and tear definition. Higher prevalence estimates were observed in symptomatic, trauma, or outpatient populations and in specific high-exposure groups than in asymptomatic or never-presented populations. The prevalence of full-thickness tears was lower than the prevalence estimated under an any-tear definition. Detailed subgroup results are presented in **Table 3**.

#### 3.3.3 Exploratory meta-regression analysis

Univariable meta-regression identified population source as the only moderator significantly associated with prevalence estimates (omnibus *P* < 0.001; pseudo-R² = 62.1%). Compared with asymptomatic or never-presented populations, prevalence estimates were higher by 31.7 percentage points (*95% CI*, 15.3%–48.1%; *P* < 0.001) in symptomatic/trauma/outpatient populations and by 33.2 percentage points (*95% CI*, 17.8%–48.6%; *P* < 0.001) in specific high-exposure groups. Publication year, imaging modality, geographic region, and AHRQ quality score were not statistically significant moderators. Logit-scale analyses yielded similar findings (**Supplementary File 3**; **Table 1**).

### 3.4 Assessment of small-study effects

**Figure 3:**
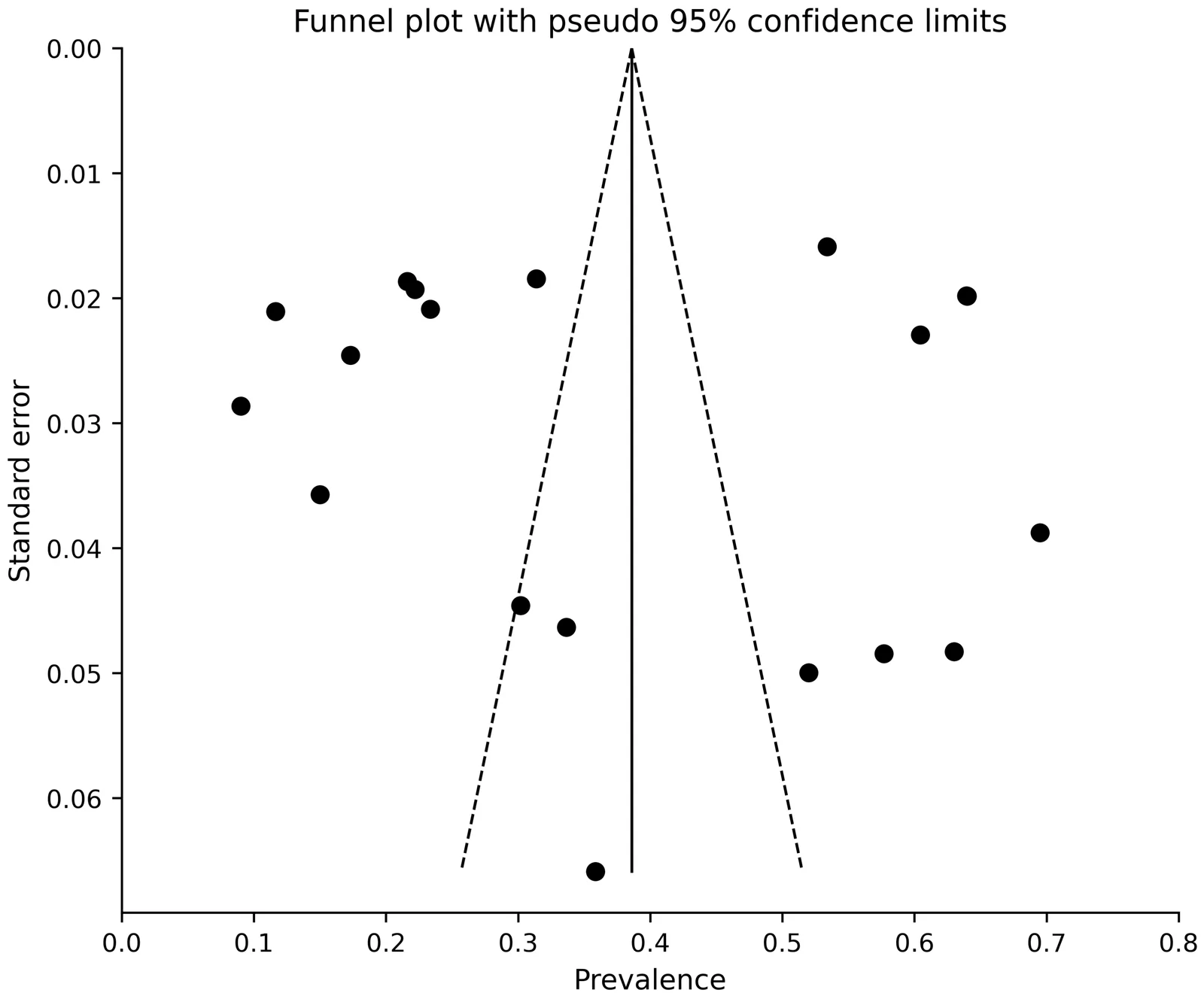
Funnel plot for exploratory assessment of small-study effects in studies of rotator cuff tear prevalence.

Visual inspection of the funnel plot indicated some asymmetry (**Figure 3**). Formal tests did not detect statistically significant small-study effects: the random-effects *Egger-type test* yielded *P* = 0.071, the traditional *Egger test* yielded *P* = 0.107, and the *Begg–Mazumdar* test yielded *P* = 0.332. These findings should be interpreted cautiously because of the substantial between-study heterogeneity (**Supplementary File 3**; **Table 3**).

### 3.5 Sensitivity analyses

**Figure 4:**
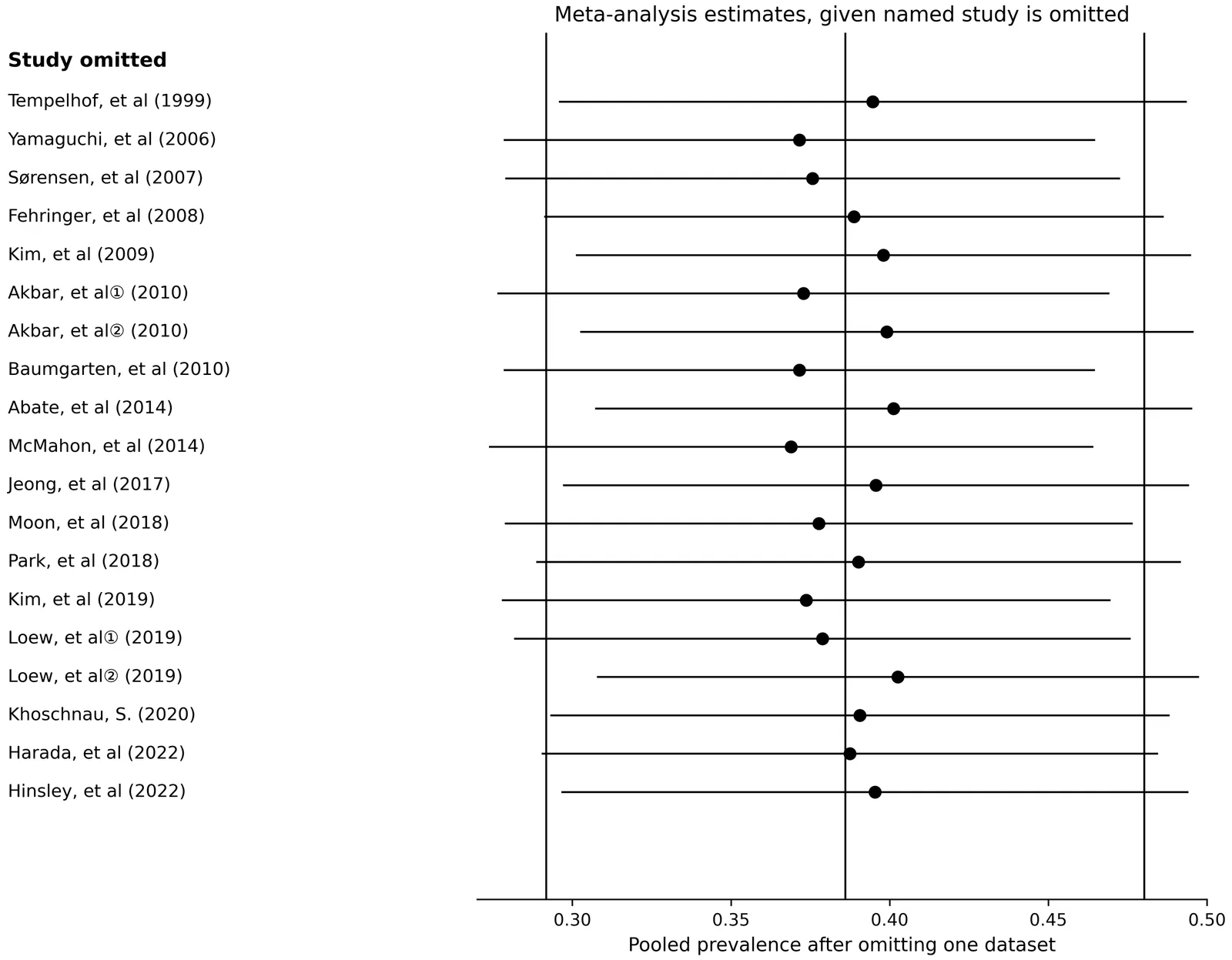
Leave-one-dataset-out sensitivity analysis of pooled rotator cuff tear prevalence.

Leave-one-dataset-out analysis did not materially alter the pooled prevalence, indicating that the summary estimate was not driven by any single dataset (**Figure 4**). Restricted analyses yielded pooled prevalence estimates of 63.5% (*95% CI*, 60.8%–66.1%) in shoulder-related clinical populations and 64.0% (*95% CI*, 61.2%–66.7%) in populations explicitly enrolled because of shoulder pain (**Supplementary File 3**; **Table 2**). Analyses using logit and *Freeman–Tukey* double-arcsine transformations, together with REML-based models, yielded comparable overall estimates, supporting the robustness of the primary findings (**Supplementary File 3**; **Table 3**).

## 4 Discussion

This systematic review and meta-analysis provide a stratified synthesis of the prevalence of rotator cuff tears across diverse research settings. The pooled prevalence was 38.6%, indicating that rotator cuff tears are commonly detected in imaging-based studies. The most consistent variation was observed across population sources, with substantially higher prevalence in symptomatic, trauma, outpatient, and specific high-exposure populations than in asymptomatic or never-presented populations. Full-thickness tears were less prevalent than estimates based on an any-tear definition. These findings demonstrate a considerable burden of rotator cuff tears while also showing that the pooled estimate reflects a mixture of distinct population settings rather than a single population-wide prevalence.

Population source emerged as the principal measured contributor to between-study heterogeneity. Participants undergoing imaging because of shoulder pain, trauma, functional impairment, or occupational exposure are more likely to have a rotator cuff tear than individuals recruited through community screening or asymptomatic cohorts (Grusky et al., 2021; Mayrhuber et al., 2022; Jahanian et al., 2023; Migliorini et al., 2023; Chiba et al., 2025). Referral pathways and selective imaging may therefore increase the pretest probability of a tear in clinical studies. Conversely, the detection of rotator cuff tears in asymptomatic individuals indicates that structural abnormalities do not necessarily correspond to symptomatic disease burden (Altamimi et al., 2024; Sanders et al., 2025; Schwitzguébel, 2026). The restricted prevalence estimates of approximately 64% in shoulder-related clinical populations further support the importance of clinical context, although these analyses were based on relatively few studies.

Prevalence generally increased with age, consistent with progressive changes in tendon structure, vascularity, repair capacity, and cumulative mechanical loading (Davis, 2023; Jain and Khazzam, 2024; Liu et al., 2024; Sripathi and Agrawal, 2024; Wang et al., 2024; Distefano et al., 2026). The absence of a strictly monotonic pattern in the oldest age groups may reflect the small number of contributing studies and differences in population composition rather than a true decline in prevalence. The higher prevalence observed in men may similarly be influenced by occupational and mechanical exposure rather than sex alone (Song et al., 2022; Yanik et al., 2023; Versloot et al., 2024). Although regional and temporal differences were observed in subgroup analyses, neither geographic region nor publication year was significantly associated with prevalence in meta-regression, suggesting that these patterns may largely reflect differences in sampling, population source, and study design.

Tear definition had a substantial influence on prevalence estimates. Studies using an any-tear definition naturally captured a broader spectrum of structural abnormalities than studies restricted to full-thickness tears, underscoring the need for consistent case definitions in epidemiological research. Ultrasound- and MRI-based studies yielded broadly similar pooled estimates, but this finding should not be interpreted as evidence of diagnostic equivalence. Ultrasound performance depends on operator expertise, equipment, and local quality assurance, whereas MRI provides more comprehensive structural characterization but is less accessible and more costly. Variation in imaging protocols and interpretation criteria may therefore continue to influence prevalence estimates (Zoga et al., 2021; Obeidat et al., 2024; Toh, 2024).

The overall findings were stable in leave-one-dataset-out analyses and under alternative effect-size transformations and variance estimators, supporting the robustness of the principal prevalence pattern. Additional strengths of this review include the search of multiple Chinese and English databases, the inclusion of diverse population settings, the use of per-person prevalence estimates, and stratified analyses across population source, age, sex, region, tear definition, and imaging modality.

Several limitations should be acknowledged. First, many included studies enrolled selected clinical or high-exposure populations rather than representative population-based samples, limiting the generalizability of the pooled estimate. Second, substantial heterogeneity remained because of differences in population characteristics, tear definitions, sampling procedures, and imaging protocols. Third, the meta-regression was based on study-level variables and univariable models, and the restricted clinical analyses included only a small number of studies; therefore, residual confounding and ecological bias remain possible. Assessment of small-study effects was also limited by the marked heterogeneity.

These findings support greater attention to rotator cuff tear prevention, risk assessment, and early identification, particularly in symptomatic individuals and populations exposed to sustained shoulder loading. Clinical assessment and imaging decisions should be guided by symptom status, exposure profile, diagnostic purpose, and local expertise rather than indiscriminate population screening. Future epidemiological studies should prioritize representative sampling, standardized tear definitions, and harmonized imaging and reporting criteria.

## 5 Conclusion

The pooled prevalence of rotator cuff tears was 38.6%, with markedly higher estimates in symptomatic/trauma/outpatient populations and specific high-exposure groups. Population source was the principal measured contributor to between-study heterogeneity, highlighting the importance of population-specific estimates for targeted risk assessment, early identification, and epidemiological planning. Future studies should prioritize representative sampling, standardized tear definitions, and harmonized imaging criteria to improve the comparability and applicability of prevalence estimates.

## Data Availability

The original contributions presented in the study are included in the article/**Supplementary Material**. Further inquiries can be directed to the corresponding author.
