# Supplementary material for: Prevalence of rotator cuff tears: a systematic review and meta-analysis of observational studies
Source: Front Physiol. 2026 Sep 3;17:1876217. doi: 10.3389/fphys.2026.1876217 (PMC13581783; doi:10.3389/fphys.2026.1876217)
Supplement: Supplementary file 1 [file DataSheet1.docx]

**Supplementary File 1. Full Search Strategies**

The literature search was conducted in PubMed, Web of Science Core Collection, Embase, the Cochrane Library, China National Knowledge Infrastructure (CNKI), Wanfang Data, and the VIP Chinese Science and Technology Journal Database from database inception to December 1, 2025. No language, publication-date, or study-design filters were applied at the search stage. Database-specific subject headings and free-text terms related to rotator cuff tears and prevalence or epidemiology were combined using Boolean operators. The reference lists of included studies and relevant reviews were also screened manually.

| **Database** | **Platform/search field** | **Search strategy** |
| --- | --- | --- |
| PubMed | NCBI PubMed; MeSH terms and Title/Abstract fields | ("Rotator Cuff"[Mesh] OR "Rotator Cuff Injuries"[Mesh] OR "rotator cuff"[tiab] OR "rotator cuff tear"[tiab] OR "rotator cuff tears"[tiab] OR "rotator cuff injury"[tiab] OR "rotator cuff injuries"[tiab] OR "rotator cuff disease"[tiab] OR "rotator cuff diseases"[tiab] OR "rotator cuff pathology"[tiab] OR "rotator cuff pathologies"[tiab]) AND ("Prevalence"[Mesh] OR "Epidemiology"[Subheading] OR prevalen*[tiab] OR epidemiolog*[tiab]) |
| Web of Science Core Collection | Clarivate Web of Science; Topic field (title, abstract, author keywords, and Keywords Plus) | TS=(("rotator cuff" NEAR/3 (tear* OR injur* OR disease* OR patholog*)) OR "rotator cuff") AND TS=(prevalen* OR epidemiolog*) |
| Embase | Elsevier Embase; Emtree terms and title/abstract/author-keyword fields | ('rotator cuff'/exp OR 'rotator cuff tear*':ti,ab,kw OR 'rotator cuff injur*':ti,ab,kw OR 'rotator cuff disease*':ti,ab,kw OR 'rotator cuff patholog*':ti,ab,kw) AND ('prevalence'/exp OR 'epidemiology'/exp OR prevalen*:ti,ab,kw OR epidemiolog*:ti,ab,kw) |
| Cochrane Library | Cochrane Library Advanced Search; MeSH descriptors and title/abstract/keyword fields | #1 MeSH descriptor: [Rotator Cuff] explode all trees; #2 MeSH descriptor: [Rotator Cuff Injuries] explode all trees; #3 ("rotator cuff" OR "rotator cuff tear*" OR "rotator cuff injur*" OR "rotator cuff disease*" OR "rotator cuff patholog*"):ti,ab,kw; #4 MeSH descriptor: [Prevalence] explode all trees; #5 (prevalen* OR epidemiolog*):ti,ab,kw; #6 (#1 OR #2 OR #3) AND (#4 OR #5) |
| CNKI | CNKI Advanced Search; Subject field | 主题 = ("肩袖撕裂" OR "肩袖损伤" OR "肩袖病变" OR "肩袖疾病") AND 主题 = ("患病率" OR "流行病学" OR "流行病学特征") |
| Wanfang Data | Wanfang Data Advanced Search; Subject field | 主题 = ("肩袖撕裂" OR "肩袖损伤" OR "肩袖病变" OR "肩袖疾病") AND 主题 = ("患病率" OR "流行病学" OR "流行病学特征") |
| VIP Database | VIP Advanced Search; Title, keyword, and abstract fields | 题名/关键词/摘要 = ("肩袖撕裂" OR "肩袖损伤" OR "肩袖病变" OR "肩袖疾病") AND 题名/关键词/摘要 = ("患病率" OR "流行病学" OR "流行病学特征") |

Additional sources. The reference lists of all included studies and relevant systematic reviews were screened manually. No additional eligible records were identified from these sources.

Abbreviations: CNKI, China National Knowledge Infrastructure; MeSH, Medical Subject Headings; TS, Topic Search.
