# Supplementary material for: Prevalence of rotator cuff tears: a systematic review and meta-analysis of observational studies
Source: Front Physiol. 2026 Sep 3;17:1876217. doi: 10.3389/fphys.2026.1876217 (PMC13581783; doi:10.3389/fphys.2026.1876217)
Supplement: Supplementary file 2 [file DataSheet2.docx]

**Supplementary File 2. Study-Level Quality Assessment Results**

Methodological quality was assessed using the 11-item Agency for Healthcare Research and Quality (AHRQ) checklist for cross-sectional studies. Each item was scored as 1 for “Yes” and 0 for “No” or “Unclear.”

| **No.** | **Study** | **Q1** | **Q2** | **Q3** | **Q4** | **Q5** | **Q6** | **Q7** | **Q8** | **Q9** | **Q10** | **Q11** | **Total** | **Quality** |
| --- | --- | --- | --- | --- | --- | --- | --- | --- | --- | --- | --- | --- | --- | --- |
| 1 | Tempelhof et al., 1999 | 1 | 1 | 1 | 1 | 1 | 1 | 1 | 0 | 1 | 1 | 0 | 9 | High |
| 2 | Yamaguchi et al., 2006 | 1 | 1 | 1 | 1 | 0 | 1 | 1 | 1 | 0 | 1 | 0 | 8 | High |
| 3 | Sørensen et al., 2007 | 1 | 1 | 1 | 1 | 1 | 1 | 1 | 0 | 1 | 1 | 1 | 10 | High |
| 4 | Fehringer et al., 2008 | 1 | 1 | 0 | 0 | 0 | 1 | 0 | 1 | 0 | 1 | 0 | 5 | Moderate |
| 5 | Kim et al., 2009 | 1 | 1 | 0 | 0 | 0 | 1 | 0 | 1 | 0 | 1 | 0 | 5 | Moderate |
| 6 | Akbar et al., 2010 | 1 | 1 | 1 | 0 | 0 | 1 | 1 | 1 | 1 | 1 | 0 | 8 | High |
| 7 | Baumgarten et al., 2010 | 1 | 1 | 1 | 1 | 1 | 1 | 0 | 1 | 1 | 1 | 0 | 9 | High |
| 8 | Abate et al., 2014 | 1 | 1 | 0 | 0 | 0 | 0 | 1 | 1 | 0 | 0 | 0 | 4 | Moderate |
| 9 | McMahon et al., 2014 | 1 | 1 | 1 | 0 | 1 | 0 | 0 | 0 | 1 | 1 | 0 | 6 | Moderate |
| 10 | Jeong et al., 2017 | 1 | 1 | 1 | 0 | 0 | 1 | 1 | 1 | 0 | 1 | 0 | 7 | Moderate |
| 11 | Moon et al., 2018 | 1 | 1 | 1 | 1 | 0 | 0 | 0 | 1 | 0 | 1 | 0 | 6 | Moderate |
| 12 | Park et al., 2018 | 1 | 1 | 0 | 1 | 1 | 0 | 1 | 1 | 1 | 1 | 0 | 8 | High |
| 13 | Kim et al., 2019 | 1 | 1 | 1 | 1 | 1 | 0 | 0 | 1 | 0 | 1 | 0 | 7 | Moderate |
| 14 | Loew et al., 2019 | 1 | 1 | 0 | 0 | 1 | 1 | 1 | 1 | 0 | 1 | 0 | 7 | Moderate |
| 15 | Khoschnau et al., 2020 | 1 | 1 | 1 | 1 | 0 | 0 | 0 | 0 | 0 | 1 | 0 | 5 | Moderate |
| 16 | Harada et al., 2022 | 1 | 1 | 0 | 0 | 1 | 1 | 1 | 0 | 0 | 1 | 0 | 6 | Moderate |
| 17 | Hinsley et al., 2022 | 1 | 1 | 1 | 1 | 0 | 1 | 1 | 1 | 1 | 1 | 1 | 10 | High |

**Scoring and classification:** Total scores of 8–11 were classified as high quality, 4–7 as moderate quality, and 0–3 as low quality. AHRQ scores were assigned at the study level; studies contributing more than one independent dataset retained a single study-level quality score.

**AHRQ Checklist Item Definitions**

| **Item** | **Definition** |
| --- | --- |
| **Q1** | Define the source of information (survey or record review). |
| **Q2** | List inclusion and exclusion criteria for exposed and unexposed participants, or refer to previous publications. |
| **Q3** | Indicate the period used for identifying participants. |
| **Q4** | Indicate whether participants were consecutive if the study was not population-based. |
| **Q5** | Indicate whether evaluators of subjective components were masked to other aspects of participant status. |
| **Q6** | Describe assessments undertaken for quality assurance purposes. |
| **Q7** | Explain participant exclusions from the analysis. |
| **Q8** | Describe how confounding was assessed and/or controlled. |
| **Q9** | Explain how missing data were handled, when applicable. |
| **Q10** | Summarize response rates and completeness of data collection. |
| **Q11** | Clarify expected follow-up and the proportion of participants with incomplete data or follow-up, when applicable. |
