# Supplementary material for: Prevalence of rotator cuff tears: a systematic review and meta-analysis of observational studies
Source: Front Physiol. 2026 Sep 3;17:1876217. doi: 10.3389/fphys.2026.1876217 (PMC13581783; doi:10.3389/fphys.2026.1876217)
Supplement: Supplementary file 3 [file DataSheet3.docx]

**Supplementary File 3. Additional Statistical Analyses**

This file presents the additional analyses supporting the main meta-analysis, including exploratory univariable meta-regression, restricted analyses by clinical population definition, sensitivity analyses using alternative prevalence transformations, and formal assessments of small-study effects. The exploratory meta-regression, restricted clinical-population analyses, alternative prevalence transformations, REML-based sensitivity analyses, and assessments of small-study effects were conducted post hoc. The primary analysis in Table 3 and the original broad clinical subgroup in Table 2 are shown for comparison.

**Table 1. Exploratory univariable meta-regression analyses**

**Panel A. Analyses on the raw prevalence scale**

| **Moderator** | **Coding / comparison** | **Datasets, n** | **Coefficient, percentage points** | **95% CI** | **P value** | **Residual tau²** | **Pseudo-R², %** |
| --- | --- | --- | --- | --- | --- | --- | --- |
| Publication year | Per 10-year increase | 19 | -3.71 | -20.24 to 12.82 | 0.641 | 0.04362 | 0.00 |
| AHRQ score | Per 1-point increase | 19 | 2.85 | -3.08 to 8.78 | 0.325 | 0.04168 | 0.03 |
| Population source | Overall categorical test | 19 | - | - | <0.001 | 0.01582 | 62.06 |
| Population source | Specific high-exposure vs asymptomatic / never-presented | - | 33.22 | 17.81 to 48.62 | <0.001 | - | - |
| Population source | Symptomatic / trauma / outpatient vs asymptomatic / never-presented | - | 31.72 | 15.35 to 48.08 | <0.001 | - | - |
| Imaging modality | MRI vs ultrasound | 19 | 3.01 | -18.25 to 24.27 | 0.769 | 0.04396 | 0.00 |
| Geographic region | Overall categorical test | 19 | - | - | 0.279 | 0.03968 | 4.83 |
| Geographic region | East Asia vs Europe | - | 9.39 | -14.53 to 33.31 | 0.418 | - | - |
| Geographic region | North America vs Europe | - | 18.52 | -5.38 to 42.42 | 0.120 | - | - |

**Note:** Coefficients are percentage-point changes or differences in prevalence. Europe, ultrasound, and asymptomatic / never-presented populations were the reference categories for categorical moderators. AHRQ, Agency for Healthcare Research and Quality; CI, confidence interval; MRI, magnetic resonance imaging. All meta-regression models used restricted maximum likelihood estimation with Knapp-Hartung adjustment. Negative pseudo-R² values were reported as 0%.

**Panel B. Logit-scale sensitivity analyses**

| **Moderator** | **Moderator P value** | **Pseudo-R², %** | **Assessment** |
| --- | --- | --- | --- |
| Publication year | 0.664 | 0.00 | Consistent with the main analysis |
| AHRQ score | 0.312 | 0.42 | Consistent with the main analysis |
| Population source | <0.001 | 60.46 | Remained statistically significant |
| Imaging modality | 0.826 | 0.00 | Consistent with the main analysis |
| Geographic region | 0.270 | 4.59 | Consistent with the main analysis |

**Table 2. Restricted sensitivity analyses according to clinical population definition**

| **Analysis** | **Included studies** | **No. of studies** | **Cases / participants** | **Pooled prevalence, % (95% CI)** | **I², %** |
| --- | --- | --- | --- | --- | --- |
| Original broad clinical subgroup | Yamaguchi; Sørensen; Fehringer; Baumgarten | 4 | 846 / 1,382 | 55.5 (45.2 to 65.8) | 92.5 |
| Restricted shoulder-related clinical population | Yamaguchi; Sørensen; Baumgarten | 3 | 811 / 1,278 | 63.5 (60.8 to 66.1) | 0.0 |
| Strict shoulder-pain population | Yamaguchi; Baumgarten | 2 | 751 / 1,174 | 64.0 (61.2 to 66.7) | 0.0 |

**Note:** The original broad subgroup included one study of older lower-limb orthopaedic outpatients and therefore was not restricted to patients presenting with shoulder symptoms. The restricted analyses included patients with shoulder pain or acute shoulder trauma, and the strict analysis was limited to studies explicitly enrolling patients because of shoulder pain. Pooled prevalence estimates were derived from meta-analytic models rather than from crude proportions calculated using aggregated case and participant counts. The original broad subgroup used a random-effects model, whereas the two restricted analyses used fixed-effect models.

**Table 3. Alternative prevalence transformations and exploratory assessment of small-study effects**

**Panel A. Sensitivity analyses using alternative transformations and heterogeneity estimators**

| **Analysis role** | **Transformation** | **tau² estimator** | **Datasets** | **Pooled prevalence, % (95% CI)** | **I², %** |
| --- | --- | --- | --- | --- | --- |
| Primary analysis | Raw proportion | DerSimonian-Laird | 19 | 38.6 (29.2 to 48.0) | 98.5 |
| Alternative transformation | Logit | DerSimonian-Laird | 19 | 36.6 (28.1 to 45.9) | 97.8 |
| Alternative transformation | Freeman-Tukey double-arcsine | DerSimonian-Laird | 19 | 37.6 (28.5 to 47.2) | 98.2 |
| Estimator sensitivity | Raw proportion | REML | 19 | 38.6 (29.3 to 47.9) | 98.5 |
| Estimator sensitivity | Logit | REML | 19 | 36.5 (27.0 to 47.1) | 97.8 |
| Estimator sensitivity | Freeman-Tukey double-arcsine | REML | 19 | 37.6 (28.2 to 47.5) | 98.2 |

**Note:** Freeman-Tukey estimates were back-transformed using the harmonic mean of study sample sizes. REML, restricted maximum likelihood.

**Panel B. Exploratory tests for small-study effects / funnel-plot asymmetry**

| **Effect scale** | **Test** | **Model / inference** | **Test parameter** | **Estimate (95% CI, where applicable)** | **P value** |
| --- | --- | --- | --- | --- | --- |
| Logit prevalence | Random-effects Egger-type meta-regression | REML + Knapp-Hartung | Slope for standard error | -5.30 (-11.09 to 0.50) | 0.071 |
| Logit prevalence | Traditional Egger regression | Multiplicative fixed-effect form | Intercept | -5.75 (-12.88 to 1.38) | 0.107 |
| Logit prevalence | Begg-Mazumdar rank correlation | Kendall rank test | Kendall's tau | -0.170 | 0.332 |

**Note:** Formal tests were interpreted in conjunction with the funnel plot and the substantial between-study heterogeneity. CI, confidence interval; REML, restricted maximum likelihood.
